# Dairy intake revisited – associations between dairy intake and lifestyle related cardio-metabolic risk factors in a high milk consuming population

**DOI:** 10.1186/s12937-018-0418-y

**Published:** 2018-11-22

**Authors:** Ingegerd Johansson, Lena Maria Nilsson, Anders Esberg, Jan-Håkan Jansson, Anna Winkvist

**Affiliations:** 1 Department of Nutritional Research, Umeå University, Umeå, Sweden; 2 Department of Odontology, Umeå University, Umeå, Sweden; 3 Department of Public Health and Clinical Medicine, Research Unit Skellefteå, Umeå University, Umeå, Sweden; 4 Department of Internal Medicine and Clinical Nutrition, Sahlgrenska Academy, University of Gothenburg, Gothenburg, Sweden

**Keywords:** Dairy products, Milk, Cheese, Butter, Fermented milk, Non-fermented milk, BMI, Serum lipids, Blood glucose, Blood pressure

## Abstract

**Background:**

The association between milk and dairy intake and the incidence of cardiometabolic diseases, cancer and mortality has been evaluated in many studies, but these studies have had conflicting results with no clear conclusion on causal or confounding associations. The present study aims to further address this association by cross-sectional and longitudinal evaluation of the associations between exposure to various types of dairy products and metabolic risk markers among inhabitants in northern Sweden while taking other lifestyle factors into account.

**Methods:**

Respondents in the Västerbotten Intervention Programme with complete and plausible diet data between 1991 and 2016 were included, yielding 124,934 observations from 90,512 unique subjects. For longitudinal analysis, 27,682 participants with a visit 8–12 years after the first visit were identified. All participants completed a validated Food Frequency Questionnaire. Metabolic risk markers, including body mass index (BMI), blood pressure, serum (S) cholesterol and triglycerides, and blood glucose, were measured. Participants were categorized into quintiles by intake of dairy products, and risk (odds ratios, OR) of undesirable levels of metabolic risk markers was assessed in multivariable logistic regression analyses. In longitudinal analyses, intake quintiles were related to desirable levels of metabolic risk markers at both visits or deterioration at follow-up using Cox regression analyses.

**Results:**

The OR of being classified with an undesirable BMI decreased with increasing quintiles of total dairy, cheese and butter intake but increased with increasing non-fermented milk intake. The OR of being classified with an undesirable S-cholesterol level increased with increasing intake of total dairy, butter and high fat (3%) non-fermented milk, whereas an undesirable S-triglyceride level was inversely associated with cheese and butter intake in women. In longitudinal analyses, increasing butter intake was associated with deterioration of S-cholesterol and blood glucose levels, whereas increasing cheese intake was associated with a lower risk of deterioration of S-triglycerides.

**Conclusions:**

Confounding factors likely contribute to the demonstrated association between dairy intake and mortality, and other medical conditions and analyses should be stratified by dairy type.

**Electronic supplementary material:**

The online version of this article (10.1186/s12937-018-0418-y) contains supplementary material, which is available to authorized users.

## Introduction

The association between milk and dairy intake and the incidence of cardiometabolic diseases, cancer and mortality has been evaluated in a large number of observational studies, meta-analyses and gene based Mendelian randomization studies [1–3]. Many, but not all, of these studies showed a null or protective effect of dairy intake. However, most studies were carried out in countries with low milk consumption concurrent with a high prevalence of lactose intolerance. In this respect, Sweden represents contrasting environmental and genetic exposure. Here, milk consumption is frequent, and only 5–7% of inhabitants carry the lactose intolerant associated C/C allele of C/T-13910 (rs4988235) or G/A-22018 (rs182549) in the MCM6 gene, which regulates lactase production by the lactase-phlorizin hydrolase (LCT) gene [4, 5]. According to FAO food balance sheets, Sweden had the fourth highest per capita milk supply worldwide in 2013 with an average of 341.2 kg per year [6]. Consequently, Sweden offers a suitable setting for research on the impact of dairy intake on human health.

To date, nine observational studies on dairy intake and health have been carried out in Sweden [7–15]. Of these, five evaluated their association with cardiovascular diseases and confirmed the null or protective effect pattern for intake of total dairy foods, specific type of dairy products or fatty acid biomarkers 15:0 and 17:0 [7–11]. In contrast, recent studies in Sweden [12–15] found an association between increasing non-fermented milk intake and total mortality. In our study [14], we also demonstrated significantly higher total mortality in subjects with high butter intake, whereas high cheese intake was associated with lower total mortality.

Importantly, the dairy types non-fermented milk, fermented milk and cheese differ in fat, lactose and bioactive peptide content with potential health effects. The deleterious health effects of high fat diets have long been known; hence, low fat milk and dairy products have been promoted as healthy and nutritious by the Nordic Nutrition Recommendations and by international recommendations [16, 17]. Accordingly, intake of high fat milk dropped markedly in favour of medium fat options from 1986 to 1991 in Sweden [18]. However, in recent years, fat intake from milk and other dairy products has increased again in Sweden, likely reflecting the strong promotion of high fat diets in Swedish media for a period [19]. Further, lactose is degraded to free galactose in the intestine, and this degradation has been associated with oxidative stress and chronic inflammation in animals [20]. Notably, bacterial scavenging of galactose in the intestine is low as galactose metabolism is not favoured in the presence of glucose, which is the case after lactose degradation. Finally, bioactive peptides have effects on the microbiota in the gastrointestinal canal, inflammatory responses and cell signalling in the host [21]. In short, the effects on health-associated events likely differ by dairy type and fat content, which has been addressed in only a few studies comparing these factors to total dairy intake [22].

Does increased all-cause mortality concurrent with the consumption of non-fermented milk and butter represent a causal association or simply residual confounding by associated lifestyle factors? The present study aims to address this question by cross-sectional and longitudinal evaluations of the associations between exposure to different types of dairy products and risk markers for cardiovascular diseases in a large cohort of inhabitants in northern Sweden while taking other lifestyle factors into account.

## Subjects and methods

### Data source and study participants

Respondents in the Västerbotten Intervention Programme (VIP) and included in the Northern Sweden Diet Database (NSDD; http://www.biobank.umu.se/biobank/northern-sweden-diet-database/?languageId=1) were eligible for the present study. The VIP runs in the county of Västerbotten in Northern Sweden with approximately 260,000 inhabitants, out of which close to 121,000 live in the main city of Umeå. Residents are invited to receive a health examination at 40, 50 and 60 years of age and, for a period, some communities also invited 30-year-olds. Thus, 10- and 20-year follow-up data are available for a subgroup. Participants in the VIP undergo an extensive health examination, including anthropometric measurements, blood pressure, serum lipid profiles, and oral glucose levels before and after a glucose tolerance test, and they answer an extensive questionnaire on diet, lifestyle, health and life conditions. The average recruitment rate has been approximately 60% of available participants. Only very limited evidence of selection bias in relation to income, age and unemployment has been reported [23], and no difference was observed in cancer incidence in the VIP cohort versus in the general population of Västerbotten [24].

**Fig. 1:**
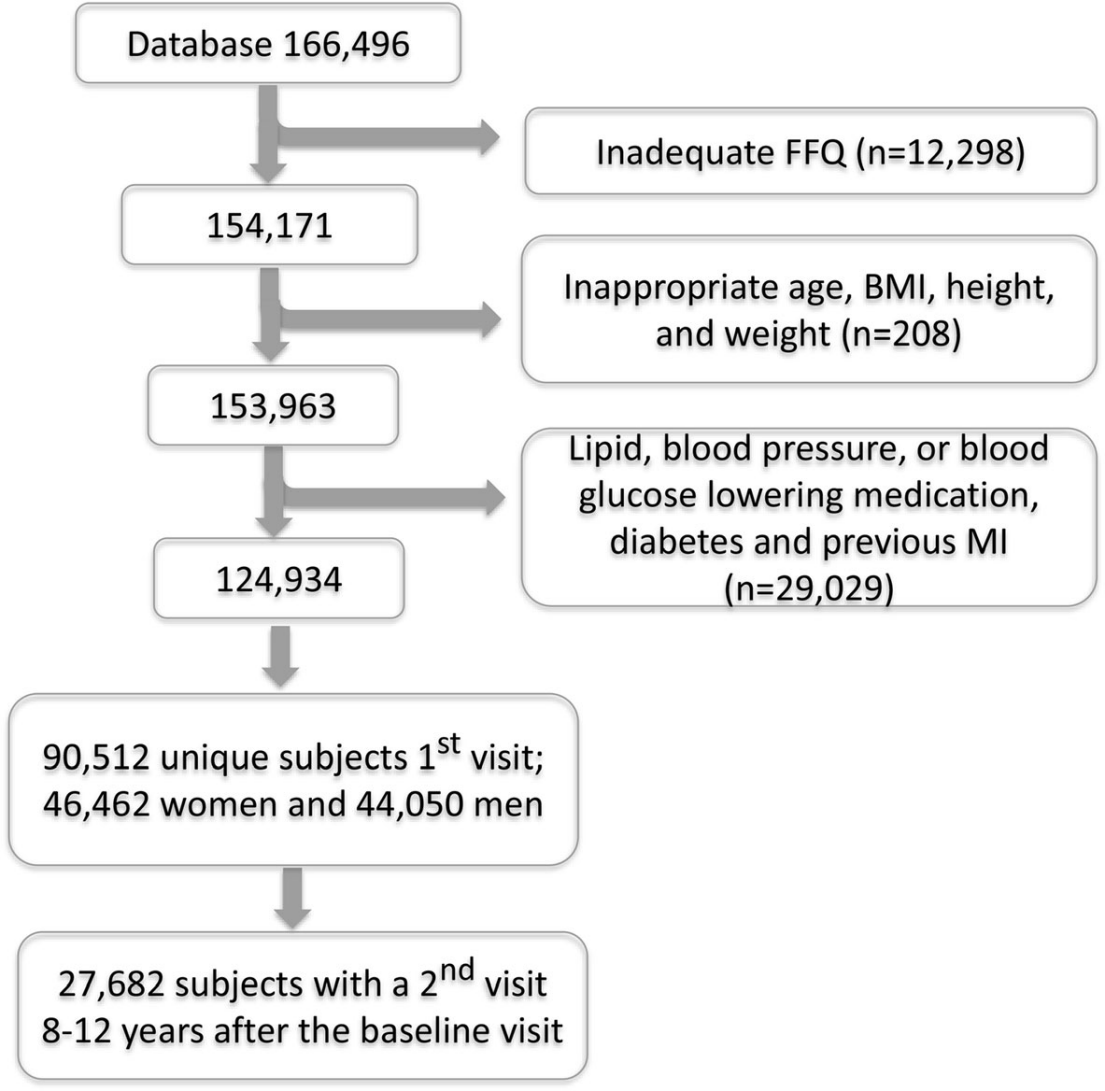
Study flow chart

The basic database included 166,496 observations (51.3% women) from Jan 1st, 1991, to December 31, 2016. For the present study, observations were excluded if (*i*) the food intake recording was incomplete, i.e., ≥10% missing data and/or a missing portion indication, extreme (highest and lowest 1%) food intake levels (FIL; [18]), and extreme energy intake (lowest 1% and > 5000 kCal); (*ii*) age < 29 years or > 65 years, implausible height (< 130 or > 210 cm) or weight (< 35 kg) values, or BMI < 15. In addition, subjects who reported intake of serum lipid, blood glucose or blood pressure lowering medication, who have diabetes or who had been hospitalized for a myocardial infarction were excluded. The final study group included 124,934 observations, 90,512 of which were unique subjects (51.3% women and 48.7% men) from the first screening event between 1991 and 2016. The flow chart is shown in Fig. 1.

### Dietary assessments

All participants completed a Food Frequency Questionnaire (FFQ) targeting their dietary habits, including beer, wine and spirits in the most recent year. Over the study period, two versions of the FFQ were used: a longer version (84 food items/aggregates) and a shortened version (64–66 food items/aggregates). The longer version, which was used until 1996, was completed by 31%, and the shorter version was completed by 69% of the participants at their first visit and 100% at follow-up visits. The shorter version was created by deleting and merging a few related food items. The questions about dairy products and beer, wine and spirits remained unchanged over the study period. In the FFQ, intake was reported on a fixed 9-level scale. Meal-time portion sizes were estimated with the support of four colour pictures of a plate containing increasing amounts of staple foods (potato/rice/pasta), main protein sources (meat/fish), and vegetables. For other foods, either sex- and age-specific portion sizes or fixed sizes, such as an apple or egg, were applied. Total estimated daily intake of energy (excluding energy from alcohol) and nutrients was calculated by weighting reported intake frequencies by food composition provided by the National Food Agency (https://www.livsmedelsverket.se/en/food-and-content/naringsamnen/livsmedelsdatabasen). Estimated intake of energy, nutrients, vitamins and minerals was previously validated against repeated 24-h dietary records and biological markers [25–28].

A diet score reflecting healthy eating habits was calculated based on rankings by sex and 10-year age groups of daily intake frequencies of four favourable food groups (fish, fruits, vegetables and whole grain foods) and four unfavourable food/beverage groups (red or processed meats, desserts and sweets, sugar-sweetened beverages and fried potatoes). The sum of all quartile ranks represents the Healthy Diet Score, which has a minimum of 0 and a maximum of 24 with higher ranks indicating healthier food and beverage choices [29].

Further, a Dietary Inflammatory Index (DII) was calculated as suggested by Shivappa and colleagues [30], but modified to fit the examined population [31]. The modified index was described previously [32].

### Assessment of metabolic markers

Body weight (kg) and height (m) were measured in participants wearing light clothes and no shoes, and BMI was calculated (body weight/height^2^). Total cholesterol (S-cholesterol) and triglycerides (S-triglycerides) were analysed in serum at the health centres using a Reflotron bench-top analyser (Boerhinger Mannheim GmbH Diagnostica, Germany) in the earliest years and using an enzymatic routine method at the Clinical Chemistry Department at the nearest local hospital after Sep 1st, 2009. Measures by the Reflotron bench-top analyser were calibrated to measures by the enzymatic routine method using algorithms created from a calibration sub-study. In addition, high density lipoprotein (S-HDL, determined enzymatically after precipitation of other lipoproteins), and low density lipoprotein (S-LDL, estimated indirectly with the Friedewald equation) were determined for all participants from 2010 onward. An oral glucose tolerance test (OGTT) following WHO standards was performed with a 75 g oral glucose load [33], and blood glucose levels (B-glucose) were analysed before and 2 h after the test using a bench-top analyser. Systolic and diastolic blood pressures were measured after 5 min of rest.

Subjects were categorized as having normal weight or not (cut-off BMI ≤ 25), optimal total cholesterol levels or not (< 5.2 mmol/l), optimal triglyceride levels or not (< 1.7 mmol/l), optimal HDL levels or not (≥1 mmol/l), optimal LDL levels or not (≤3 mmol/l), or optimal fasting blood glucose levels or not (< 6.1 mmol/l), and optimal blood pressure levels or not (systolic blood pressure < 130 mmHg or diastolic blood pressure < 80 mmHg) [34].

### Assessment of potential confounding factors

Information on tobacco use (smoking and Swedish snus (snuff)), highest level of education, and physical activity in leisure time was collected from the questionnaires. Smoking and use of Swedish snus were categorized as never used, past daily or occasional use, or present daily or occasional use. For education, participants were categorized into four levels with academic education as the highest level, and for physical activity into five levels reflecting from inactive to active. Intakes of alcohol (gram/day), fruit and vegetables (servings/day), and non-alcohol energy were estimated from the FFQ.

### Statistical analyses

Respondents were categorized into quintile groups based on their reported intake (servings/day) of total dairy products, non-fermented milk, fermented milk, cheese and butter by ranking within sex and 10-year age strata.

Quantitative measures are presented as the means [95% confidence limits (CI)] after confirmation of normality by the Kolmogorov-Smirnov test. Means were adjusted for sex, age, BMI and screening year using general lineal modelling (GLM). Discrete variables are presented as frequencies and percentages. Differences between group means/numbers were not tested since large groups led to statistically significant differences even when the differences were interpreted as biologically irrelevant.

Bivariable logistic regression was used to calculate odds ratios (OR) and corresponding 95% confidence limits (CI) of having undesirable values for total S-cholesterol, S-triglycerides, S-HDL, S-LDL, fasting B-glucose levels and blood pressure using the cut-off values described above. These models were run separately for men and women. The crude model included dairy quintile and age (and for cholesterol and triglycerides, period of analyses, i.e., before or after Sept 1st, 2009). In the full model, education, physical activity, smoking, BMI (except models with weight-associated outcome), reported alcohol, fruit and vegetable and energy intake, and screening year were added.

Participants with a follow-up screening 8–12 years after the baseline visit and with data that met the quality criteria were identified (*n* = 27,682; Fig. 1). Subjects with a desirable value for total S-cholesterol, S-triglycerides, S-HDL, S-LDL, fasting B-glucose or blood pressure at baseline and whose levels remained desirable over the follow-up period and patients who had a desirable level at baseline and were classified with an undesirable level at follow-up were included for prospective risk analysis by Cox proportional hazards regression models. Time between visits was the underlying time metric to estimate hazard ratios (HR) with 95% CI for the associations between total dairy intake/dairy type and an impairment of the medical variables, i.e., having a desirable level at baseline and an undesirable level 8–12 years later. The models included quintiles of dairy intake, sex, age at follow-up, education, smoking, year at follow-up, BMI at follow-up, physical activity at baseline and follow-up, and mean intake of alcohol, fruits and vegetables and energy over the follow-up period. All baseline measures were made before Sept 1st, 2009, but follow-up partly occurred later and therefore models on impairments of cholesterol and triglyceride status included period of analysis as a covariate. Dairy product exposure corresponded to the mean reported intake over the follow-up period to account for eventual changes over the follow-up period. The proportional hazards assumption was assured by the Schoenfeld test [35].

Partial least square multivariate modelling (PLS) was used to characterize subjects who reported the highest versus lowest intake of butter products and subjects who reported exclusive consumption of high (3%), medium (1.5%) or low (0.5%) fat non-fermented milk. The models included all 66 FFQ items (except items used for calculating the respective dependent variables) as the block of independent variables. Circos plots were used to illustrate the food selection pattern for the respective butter and milk groups based on standardized mean intake of foods with a PLS loading value (VIP) > 1.00.

Statistical analyses were performed with SPSS version 25 (IBM; SPSS Software), SAS version 9.4 and SIMCA P+ (Umetrics, Sartorius Stedim Biotech). All tests were two-sided, and *P* < 0.05 was considered statistically significant.

## Results

### Characteristics of study participants by sex and age

Characteristics of the study participants (46,462 women and 44,050 men) are presented in Table 1. Every third to more than every second participant had an undesirable (untreated) blood pressure, S-cholesterol, S-LDL or BMI, whereas every 10th participant had an undesirable (untreated) fasting blood glucose level. A higher proportion of men than women had undesirable levels of serum lipids and blood pressure.

**Table 1:** Characteristics for the 29–65 year old women (*n* = 46,462) and men (*n* = 44,050) at recruitment to the study

	Women			Men		
	N	mean (95% CI) or proportion (%)	% classified as undesirable	N	mean (95% CI) or proportion (%)	% classified as undesirable
BMI, kg/m^2^	46,462^a^	25.2 (25.2, 25.2)	42.9% (BMI > 25)	44,050	26.2 (26.1, 26.2)	59.2% (BMI > 25)
S-Cholesterol, mmol/l	46,293^a^	5.55 (5.54, 5.56)	60.8% (≥5.2 mmol/l)	43,900	5.72 (5.71, 5.73)	68.4% (≥5.2 mmol/l)
S-Triglycerides, mmol/l	43,938^a^	1.05 (1.04,1.05)	2.0% (≥1.7 mmol/l)	38,230	1.19 (1.18, 1.19)	7.1% (≥1.7 mmol/l)
S-HDL, mmol/l^b^	8,398^b^	1.55 (1.54, 1.56)	4.6% (< 1.0 mmol/l)	8496	1.29 (1.28, 1.30)	18.8% (< 1.0 mmol/l)
S-LDL, mmol/l^b^	8,369^b^	3.13 (3.11, 3.15)	48.3% (> 3.0 mmol/l)	8287	3.54 (3.52, 3.56)	70.0% (> 3.0 mmol/l)
B-Glucose 0 h, mmol/l	46,294^a^	5.32 (5.31, 5.33)	10.4% (≥6.1 mmol/l)	43,899	5.40 (5.39, 5.40)	13.2% (≥6.1 mm9 l/l)
Systolic blood pressure, mmHg	46,101^a^	121.2 (121.0, 121.3)	21.8% (≥130 mmHg)	43,783	126.6 (126.5, 126.7)	30.2% (≥130 mmHg)
Diastolic blood pressure, mmHg	46,082^a^	75.5 (75.5, 75.6)	24.7% (≥80 mmHg)	43,766	79.5 (79.4, 79.6)	37.8% (≥80 mmHg)
High blood pressure	44,082^a^		31.1% (dbp or sbt ↑)	43,766		44.9% (dbp or sbt ↑)
Education, academic level, %	46,195^a^	35.7		43,858	26.2	
Smoking, present smoker, %	46,105^a^	21.0		43,447	19.1	
Swedish snuff, present user, %	43,908^a^	7.9		42,631	27.8	
Inactive at leisure time, %	45,846^a^	17.0		43,433	17.7	
Total fat intake, E%	46,462^a^	34.3 (34.2, 34.3)		44,050	37.8 (37.8, 37.9)	
Saturated fat intake, E%	46,462^a^	14.3 (14.2, 14.3)		44,050	16.0 (15.9, 16.0)	
Protein intake, E%	46,462^a^	15.2 (15.2, 15.2)		44,050	14.6 (14.6, 14.7)	
Carbohydrate intake, E%	46,462^a^	49.8 (49.8, 49.9)		44,050	46.5 (46.4, 46.6)	
Sucrose, E%	46,462^a^	6.56 (6.53, 6.59)		44,050	6.39 (6.36, 6.42)	

Age adjusted means (95% CI limits) are presented for continuous variables and percent for categorical variables. Proportions (%) classified with undesirable levels are given with cut-off limits in parentheses

*Abbreviations*: *BMI* body mass index, *LDL* low density lipoprotein, *HDL* high density lipoprotein, *E%* energy in per cent of total energy

^a^Data were collected from 1991 through 2016 and represent subjects without serum lipid, blood pressure or blood glucose lowering medication and no self-reported diabetes or myocardial infarction

^b^Data are from 2010 to 2016 when HDL and LDL were estimated in all participants. Before 2010 HDL and LDL were performed at risk indications. In total, HDL was measured in 18,240 women and 18,915 men, and LDL in 11,520 women and 11,978 men. The differences between all measurements and those after 2010 in mean values or proportions with undesirable values were modest

Generally, the intake of all dairy products increased by 10-year age groups and was higher in men than in women (Additional file 1). The difference was mainly driven by differences in high fat non-fermented milk and butter intake, whereas intake of other dairy products was more stable between the sexes and 10-year age groups (Additional file 1).

### Cross-sectional associations between dairy intake and cardiovascular risk factors

The OR to be classified with undesirable BMI, S-cholesterol, or S-triglycerides by dairy product intake are presented in Table 2 and are presented in Additional files 2, 3, 4 and 5 for blood glucose, blood pressure, S-HDL and S-LDL. In both women and men, the OR to be classified with undesirable BMI accounting for potential confounders decreased with increasing intake quintiles of total dairy product, cheese and butter, whereas it increased with increasing intake quintiles of non-fermented milk. The OR to be classified with undesirable S-cholesterol increased with increasing intake quintile of butter and total dairy products. Further, in women, the OR to be classified with undesirable S-triglyceride was inversely associated with intake quintiles of butter in women and men and cheese in women (Table 2).

**Table 2:** Odds ratio (95% CI) from multivariable logistic regression by dairy food type

	Sex	Crude model^a^				Adjusted model^b^			
		Q2	Q3	Q4	Q5	Q2	Q3	Q4	Q5
BMI									
Dairy products	W	0.87 (0.82, 0.92)^< 0.001^	0.83 (0.78, 0.88)^< 0.001^	0.75 (0.71,0.80)^< 0.001^	0.70 (0.66, 0.74)^< 0.001^	0.89 (0.82, 0.95)^0.001^	0.0.85 (0.79, 0.92)^< 0.001^	0.71 (0.66, 0.77)^< 0.001^	0.66 (0.61, 0.72)^< 0.001^
	M	0.87 (0.82, 0.93)^< 0.001^	0.80 (0.75, 0.85)^< 0.001^	0.71 (0.67, 0.76)^< 0.001^	0.71 (0.67, 0.75)^< 0.001^	0.87 (0.81, 0.94)^< 0.001^	0.0.82 (0.76, 0,89)^< 0.001^	0.73 (0.67, 0.79)^< 0.001^	0.72 (0.66, 0.79)^< 0.001^
Non-fermented milk	W	1.06 (1.00, 1.13)^0..044^	1.03 (0.97,1.09)	1.05 (0.99, 1.12)	1.15 (1.08, 1.21)^< 0.001^	1.14 (1.06, 1.23)^0.001^	1.06 (0.98, 1.14)	1.20 (1.11, 1.30)^< 0.001^	1.25 (1.15,1.35)^< 0.001^
	M	1.08 (1.01, 1.14)^0.019^	1.01 (0.95, 1.07)	0.98 (0.92, 1.04)	1.07 (1.01, 1.14)^0.028^	1.11 (1.03, 1.20)^0.006^	1.06 (0.98, 1.14)	1.11 (1.03, 1.19)^0.008^	1.20 (1.11,1.30)^< 0.001^
Fermented milk	W	1.05 (0.99, 1.12)	0.98 (0.92, 1.04)	0.91 (0.85, 0.96)^< 0.001^	0.91 (0.86, 0.97)^0.002^	1.09 (1.02, 1.18)^0.017^	1.08 (1.00, 1.17)^0,043^	1.03 (0.96, 1.11)	0.98 (0.91, 1.06)
	M	1.04 (0.98, 1.10)	0.98 (0.92, 1.04)	0.87 (0.82, 0.93)^< 0.001^	0.78 (0.73, 0.83)^< 0.001^	1.04 (0.96, 1.12)	1.07 (0.99, 1.15)	0.96 (0.89, 1.04)	0.92 (0.85, 0.99)^0.035^
Cheese	W	1.00 (0.95, 1.06)	0.84 (0.79, 0.88)^< 0.001^	0.81 (0.76, 0.86)^< 0.001^	0.69 (0.65, 0.73)^< 0.001^	1.04 (0.97, 1.11)	0.88 (0.82, 0.95)^0.001^	0.88 (0.82, 0.95)^0.001^	0.71 (0.65, 0.77)^< 0.001^
	M	1.03 (0.97, 1.09)	0.94 (0.89, 1.00)^0.059^	0.83 (0.78, 0.88)^< 0.001^	0.80 (0.75, 0.85)^< 0.001^	1.03 (0.96, 1.11)	0.93 (0.86, 1,00)	0.87 (0.80, 0.94)^< 0.001^	0.90 (0.83, 0.98)^0.013^
Butter	W	0.88 (0.83, 0.93)^< 0.001^	0.91 (0.86, 0.97) ^0.002^	0.82 (0.78, 0.87)^< 0.001^	0.68 (0.64, 0.72)^< 0.001^	0.82 (0.76, 0.89)^< 0.001^	0.84 (0.78, 0.90)^< 0.001^	0.75 (0.70, 0.81)^< 0.001^	0.60 (0.57, 0.65)^< 0.001^
	M	1.06 (1.00, 1.13)^0.060^	0.99 (0.93, 1.05)	0.78 (0.73, 0.82)^< 0.001^	0.80 (0.75, 0.85)^< 0.001^	1.03 (0.93, 1.08)	0.85 (0.79, 0.92)^< 0.001^	0.71 (0.65, 0.77)^< 0.001^	0.72 (0.67, 0.78)^< 0.001^
S-Cholesterol									
Dairy products	W	0.99 (0.93, 1.05)	1.04 (0.98, 1.11)	1.07 (1.00, 1.13) ^0.045^	1.12 (1.05, 1.19)^< 0.001^	1.05 (0.97, 1.14)	1.10 (1.02, 1.19)^0.020^	1.11 (1.03, 1.21)^0.011^	1.20 (1.09, 1.31)^< 0.001^
	M	1.02 (0.96, 1.09)	1.06 (0.99, 1.13)	1.11 (1.04, 1.18)^0.001^	1.07 (1.01, 1.14)^0.032^	1.06 (0.98, 1.14)	1.11 (1.03, 1.20)^0.007^	1.16 (1.06, 1.25)^0.001^	1.22 (1.11, 1.34)^< 0.001^
Non-fermented milk	W	0.99 (0.93, 1.06)	1.01 (0.95, 1.07)	0.95 (0.89, 1.01)	1.01 (0.95, 1.07)	1.02 (0.94, 1.10)	1.00 (0.93, 1.08)	0.98 (0.90, 1.07)	1.00 (0.92, 1.09)
	M	1.03 (0.97, 1.10)	1.06 (0.99, 1.12)	1.02 (0.96, 1.09)	1.05 (0.98, 1.11)	1.04 (0.97, 1.12)	1.09 (1.01, 1.18)^0.029^	1.10 (1.02, 1.19)^0.012^	1.05 (0.96, 1.14)
Fermented milk	W	1.01 (0.95, 1.08)	1.01 (0.95, 1.08)	0.92 (0.86, 0.98)^0.006^	0.86 (0.81, 0.91)^< 0.001^	1.02 (0.95, 1.11)	1.07 (0.99, 1.16)	0.99 (0.92, 1.08)	0.96 (0.88, 1.04)
	M	1.07 (1.01, 1.14) ^0.030^	1.00 (0.94, 1.06)	0.92 (0.87, 0.98)^0.015^	0.81 (0.76, 0.86)^< 0.001^	1.12 (1.04, 1.21)^0.004^	1.05 (0.97, 1.13)	1.03 (0.95, 1.12)	0.95 (0.88, 1.03)
Cheese	W	1.00 (0.94, 1.06)	1.05 (0.98, 1.11)	1.11 (1.05, 1.19)^0.001^	1.10 (1.03, 1.17)^0.004^	1.01 (0.94, 1.09)	1.04 (0.96, 1.12)	1.09 (1.00, 1.18)^0.044^	1.02 (0.94, 1.12)
	M	1.11 (1.04, 1.18)^< 0.001^	1.07 (1.00, 1.14)^0.042^	1.04 (0.98, 1.11)	1.04 (0.98, 1.11)	1.11 (1.03, 1.19)^0.008^	1.05 (0.97, 1.13)	1.02 (0.94, 1.10)	1.01 (0.93, 1.09)
Butter	W	1.03 (0.97, 1.09)	1.07 (1.01, 1.14)^0.024^	1.16 (1.09, 1.23)^< 0.001^	1.21 (1.14, 1.29)^< 0.001^	1.09 (1.00, 1.18)^0.047^	1.14 (1.05, 1.23)^0.001^	1.20 (1.11, 1.30)^< 0.001^	1.31 (1.21, 1.43)^< 0.001^
	M	0.99 (0.93, 1.06)	1.13 (1.06, 1.20)^< 0.001^	1.20 (1.13, 1.27)^< 0.001^	1.15 (1.08, 1.23)^< 0.001^	1.05 (0.97, 1.13)	1.18 (1.10, 1.28)^< 0.001^	1.23 (1.14, 1.34)^< 0.001^	1.27 (1.17, 1.38)^< 0.001^
S-Triglycerides									
Dairy products	W	1.01 (0.92, 1.10)	0.95 (0.87, 1.04)	0.93 (0.85, 1.02)	0.93 (0.85, 1.02)	1.07 (0.96, 1.20)	0.96 (0.85, 1.08)	0.85 (0.75, 0.97)^0.013^	0.92 (0.79, 1.06)
	M	0.96 (0.90, 1.03)	0.94 (0.88, 1.01)	0.89 (0.83, 0.95)^0.001^	0.86 (0.81, 0.93)^< 0.001^	1.04 (0.96, 1.14)	1.05 (0.96, 1.15)	0.99 (0.90, 1.09)	0.99 (0.89, 1.10)
Non-fermented milk	W	1.00 (0.91, 1.10)	1.03 (0.94, 1.13)	1.02 (0.92, 1.12)	1.19 (1.09, 1.30)^< 0.001^	1.06 (0.94, 1.19)	0.98 (0.87, 1.11)	1.00 (0.87, 1.14)	1.06 (0.94, 1.20)
	M	1.04 (0.97, 1.11)	0.96 (0.89, 1.03)	1.00 (0.93, 1.07)	1.08 (1.00, 1.16) ^0.039^	1.05 (0.96, 1.14)	1.04 (0.95, 1.14)	1.09 (1.00, 1.19)	1.11 (1.00, 1.22)
Fermented milk	W	0.91 (0.84, 1.00)^0.041^	0.88 (0.81, 0.96)^0.005^	0.77 (0.71, 0.85)^< 0.001^	0.85 (0.78, 0.92)^< 0.001^	0.97 (0.86, 1.09)	1.01 (0.90, 1.14)	0.87 (0.77, 0.99)^0.031^	0.97 (0.85, 1.09)
	M	0.99 (0.93, 1.06)	0.92 (0.86, 0.99)^0.028^	0.84 (0.78, 0.90)^< 0.001^	0.72 (0.67, 0.77)^< 0.001^	1.04 (0.95, 1.13)	1.04 (0.95, 1.14)	1.03 (0.94, 1.13)	0.95 (0.86, 1.04)
Cheese	W	0.88 (0.81, 0.96)^0.003^	0.86 (0.79, 0.94)^0.001^	0.85 (0.78, 0.93)^< 0.001^	0.81 (0.75, 0.89)^< 0.001^	0.85 (0.76, 0.95)^0.003^	0.91 (0.81, 1.02)	0.87 (0.77, 0.98)^0.026^	0.77 (0.67, 0.89)^< 0.001^
	M	1.01 (0.94, 1.08)	0.96 (0.90, 1.04)	0.85 (0.79, 0.91)^< 0.001^	0.87 (0.81, 0.94)^< 0.001^	1.04 (0.96, 1.14)	1.00 (0.92, 1.09)	0.93 (0.85, 1.02)	0.93 (0.85, 1.03)
Butter	W	0.84 (0.77, 0.92)^< 0.001^	0.82 (0.76, 0.90)^< 0.001^	0.83 (0.76, 0.91)^< 0.001^	0.80 (0.73, 0.87)^< 0.001^	0.88 (0.78, 0.99)^0.031^	0.88 (0.78, 0.98)^0.025^	0.86 (0.76, 0.97)^0.012^	0.81 (0.71, 0.92)^0.001^
	M	0.93 (0.86, 1.00) ^0.038^	0.98 (0.91, 1.05)	0.90 (0.84, 0.97)^0.005^	0.85 (0.79, 0.92)^< 0.001^	0.95 (0.87, 1.03)	1.00 (0.92, 1.09)	0.92 (0.84, 1.01)	0.89 (0.81, 0.98)^0.017^

*Abbreviations*: *W* women, *M* men

Odds ratio (95% CI) are from multivariable logistic regression for being classified with an undesirable level of BMI, cholesterol or triglycerides and increasing quintile groups (Q1 to Q5) for intake of dairy products. Q1, lowest intake, is the reference category. Statistically significant *p*-values are in superscript

^a^The crude models included age and dairy type

^b^The adjusted models also included screening year, education, BMI, physical activity, smoking, intakes of fruits and vegetables, alcohol and non-alcohol energy. The models with overweight did not include BMI, and models including S-cholesterol or S-triglyceride were also adjusted for if the analyses were performed before or after the change of analyses methods

Having an undesirable level of fasting B-glucose was inversely associated with intake quintiles of butter for men and cheese for women (Additional file 2). In both women and men, the OR for an undesirable blood pressure decreased with increasing intake quintiles of fermented milk (Additional file 3). Concurrently, the OR for an undesirable low level of S-HDL decreased with increasing intake quintiles of total dairy products, cheese and butter in men (Additional file 4). Finally, the OR to have undesirably high levels of S-LDL increased with increasing intake quintiles of butter in both women and men (Additional file 5).

### Cross-sectional associations between non-fermented milk types and cardiovascular risk factors

As a next step, we identified individuals who reported that they only consumed one type of non-fermented milk, i.e., milk with low (0.5%, *n* = 5237), medium (1.5%, *n* = 25,667) or high (3%, *n* = 5352) fat content. These individuals were categorized into regular consumers (≥1/day) or non-regular consumers (< 1/day but excluding those who never consumed the milk type). The OR to be overweight was significantly lower among both middle and high fat non-fermented milk consumers than among low fat non-fermented milk consumers. In contrast, consumers of middle and high fat non-fermented milk had significantly higher OR to have undesirable serum levels of total S-cholesterol and S-LDL than the low fat non-fermented milk consumers (Table 3).

**Table 3:** Multivariable logistic regression by fat content of consumed non-fermented milk

	Non-fermented milk			*p*-value
	0.5% fat *n* = 5237	1.5% fat *n* = 25,667	3% fat *n* = 5352	ANOVA
OR (95% CI)^a^				
BMI	1.00	0.63 (0.58, 0.69)^< 0.001^	0.52 (0.47, 0.57)^< 0.001^	
S-Cholesterol	1.00	1.10 (1.01, 1.20)^0.025^	1.13 (1.02, 1.26)^0.026^	
S-Triglycerides	1.00	0.96 (0.87, 1.08)	0.90 (0.79, 1.04)	
S-HDL^b^	1.00	1.13 (0.84, 1.53)	1.30 (0.92, 1.83)	
S-LDL^b^	1.00	1.22 (1.01, 1.46)^0.039^	1.40 (1.12, 1.74)^0.003^	
B-Glucose	1.00	0.89 (0.79, 1.00)^0.042^	0.96 (0.83, 1.11)	
Blood pressure	1.00	1.04 (0.96, 1.14)	0.92 (0.82, 1.03)	
Adjusted mean (95% CI)				
BMI	26.8 (26.7, 30.0)	25.8 (25.8, 25.9)	25.3 (25.2, 25.5)	< 0.001
S-Cholesterol	5.36 (5.32, 5.39)	5.42 (5.41, 5.44)	5.47 (5.44, 5.52)	< 0.001
S-Triglycerides	1.27 (1.25, 1.30)	1.27 (1.26, 1.28)	1.26 (1.24, 1.29)	0.841
S-HDL^b^	1.42 (1.40, 1.45)	1.43 (1.42, 1.44)	1.43 (1.41, 1.45)	0.872
S-LDL^b^	3.26 (3.19, 3.33)	3.33 (3.31, 3.36)	3.43 (3.38, 3.48)	< 0.001
B-Glucose	5.43 (5.40, 5.46)	5.39 (5.37, 5.40)	5.40 (5.38, 5.43)	0.013
Systolic blood pressure	123 (123, 124)	124 (123, 124)	124 (123, 124)	0.964
Diastolic blood pressure	77.7 (77.3, 78,0)	77.7 (77.5, 77.8)	77.4 (77.1, 77.7)	0.316
Healthy Diet Score	13.0 (12.9, 13.1)	11.8 (11.7, 11.8)	11.4 (11.3, 11.5)	< 0.001
DII-score	0.79 (0.75, 0.84)	1.15 (1.13, 1.17)	1.38 (1.33, 1.43)	< 0.001

*Abbreviations*: *BMI* body mass index, *LDL* low density lipoprotein, *HDL* high density lipoprotein, *DII* dietary inflammation index

OR (95% CI with statistically significant *p*-values in superscript) from multivariable logistic regression for being categorized with an undesirable level of BMI, cholesterol (total, HDL or LDL) or triglycerides and increasing intake of non-fermented milk of different fat content in subjects reporting exclusive consumption of one milk type. Low fat milk is the reference category. The lower section shows sex and age adjusted means for the same variables and scores for the Healthy Diet Score and the DII-score. S-cholesterol and S-triglycerides were also adjusted for if the analyses were performed before or after the change of analyses methods

^a^Fully adjusted models, including cheese type, sex, age, screening year, education, BMI, physical activity, smoking, intakes of fruits and vegetables, alcohol and non-alcohol energy, are presented. The models with overweight did not include BMI. Models including S-cholesterol or S-triglyceride were also adjusted for whether the analyses were performed before or after the change of laboratory analysis method. Interactions with gender was tested and found non-significant

^b^Subjects participating 2010 and later; *n* = 651, 5185 and 1141, respectively

In line with the results from the logistic regression, consumers of low fat non-fermented milk had the highest BMI but the lowest total S-cholesterol and S-LDL, and more favourable healthy diet and DII scores than the other consumers (Table 3).

### Cross-sectional associations between cheese types and cardiovascular risk factors

Individuals who reported consuming 28% or more fat containing cheese only (*n* = 6805) exhibited lower OR to have undesirable BMI, S-triglycerides but higher OR to have undesirable fasting blood glucose levels, than those who reported consuming cheese with 10–17% fat (*n* = 9476) only (Tables 4). Though statistically significant for most cardio-metabolic risk factors and the Healthy Diet Score, the differences for the adjusted means in consumers of 10–17% fat cheese versus 28% + fat cheese were moderate (Table 4). The exception was the higher score for the inflammatory index in high fat cheese consumers (Table 4).

**Table 4:** Multivariable logistic regression by fat content of consumed cheese

	Cheese		*p*-value
	10–17% fat *n* = 9476	≥28% fat *n* = 6805	t-test
OR (95% CI)^a^			
BMI	1.00	0.87 (0.84, 0.90)^< 0.001^	
S-Cholesterol	1.00	0.99 (0.95, 1.03)	
S-Triglycerides	1.00	0.95 (0.90, 1.00)^0.047^	
S-HDL^b^	1.00	0.99 (0.89, 1.10)	
S-LDL^b^	1.00	1.05 (0.98, 1.12)	
B-Glucose	1.00	1.07 (1.01, 1.13)^0.030^	
Blood pressure	1.00	0.97 (0.93, 1.02)	
Adjusted mean (95% CI)			
BMI	25.7 (25.7, 25.8)	25.4 (25.4, 25.5)	0.001
S-Cholesterol	5.49 (5.48, 5.50)	5.46 (5.44, 5.47)	< 0.001
S-Triglycerides	1.31 (1.30, 1.31)	1.29 (1.28, 1.30)	< 0.001
S-HDL^b^	1.41 (1.41, 1.42)	1.43 (1.42, 1.44)	0.001
S-LDL^b^	3.33 (3.31, 3.35)	3.35 (3.33, 3.36)7	< 0.001
B-Glucose	5.36 (5.35, 5.36)	5.34 (5.33, 5.36)	< 0.001
Systolic blood pressure	123.9 (123.8, 124.0)	123.6 (123.5, 123.9)	< 0.001
Diastolic blood pressure	77.4 (77.3, 77.5)	77.5 (77.4, 77.6)	0.141
Healthy Diet Score	12.0 (12.0, 12.1)	11.8 (11.7, 11.8)	0.001
DII-score	0.87 (0.85, 0.88)	1.19 (1.16, 1.21)	0.006

*Abbreviations*: *BMI* body mass index, *LDL* low density lipoprotein, *HDL* high density lipoprotein, *DII* dietary inflammation index

OR (95% CI with statistically significant *p*-values in superscript) from multivariable logistic regression for being categorized with an undesirable level of BMI, cholesterol (total, HDL or LDL) or triglycerides and increasing intake of cheese of different fat content in subjects reporting exclusive consumption of one cheese type. Low fat cheese is the reference category. The lower section shows sex and age adjusted means for the same variables and scores for the Healthy Diet Score and the DII-score. S-cholesterol and S-triglycerides were also adjusted for if the analyses were performed before or after the change of analyses methods

^a^Fully adjusted models, including cheese type, sex, age, screening year, education, BMI, physical activity, smoking, intakes of fruits and vegetables, alcohol and non-alcohol energy, are presented. The models with overweight did not include BMI. Models including S-cholesterol or S-triglyceride were also adjusted for whether the analyses were performed before or after the change of laboratory analysis method. Interactions with gender was tested and found non-significant

^b^Subjects participating 2010 and later; *n* = 651, 5185 and 1141, respectively

**Table 5:** Hazard ratio (95% CI) for developing an undesirable level of metabolic risk markers by dairy food type

	Hazard ratios of adjusted model^a,b^			
	Q2	Q3	Q4	Q5
BMI				
Dairy products	0.86 (0.75, 0.99)^0.037^	1.01 (0.88, 1.16)	0.97 (0.84, 1.13)	0.90 (0.76, 1.07)
Non-fermented milk	0.92 (0.80, 1.05)	0.95 (0.83, 1.09)	1.00 (0.87, 1.15)	0.92 (0.79, 1.07)
Fermented milk	1.03 (0.90. 1.18)	0.93 (0.81, 1.07)	0.91 (0.79, 1.05)	0.95 (0.82, 1.10
Cheese	1.01 (0.89, 1.15)	1.04 (0.91, 1.19)	0.98 (0.85, 1.13)	1.00 (0.68, 1.16)
Butter	0.99 (0.86, 1.15)	0.97 (0.84, 1.12)	1.00 (0.86, 1.16)	1.06 (0.91, 1.23)
S-Cholesterol				
Dairy products	1.04 (0.93, 1.16)	1.00 (0.90, 1.12)	1.01 (0.90, 1.14)	0.98 (0.86, 1.12)
Non-fermented milk	0.99 (0.89, 1.09)	1.01 (0.91, 1.12)	0.96 (0.86, 1.07)	0.92 (0.82, 1.03)
Fermented milk	1.01 (0.91, 1.13)	1.00 (0.90, 1.11)	1.00 (0.89, 1.11)	0.96 (0.87, 1.09)
Cheese	1.01 (0.91, 1.12)	1.03 (0.93, 1.14)	0.97 (0.87, 1.09)	0.92 (0.81, 1.03)
Butter	1.08 (0.96, 1.21)	1.13 (1.01, 1.26)^0.042^	1.16 (1.03, 1.30)^0.012^	1.12 (0.99, 1.26)^0.064^
S-Triglycerides				
Dairy products	0.93 (0.76, 1.14)	0.93 (0.76, 1.14)	0.85 (0.68, 1.06)	0.93 (0.72, 1.19)
Non-fermented milk	0.92 (0.75, 1.13)	1.02 (0.83, 1.25)	1.03 (0.84, 1.26)	1.06 (0.86, 1.32)
Fermented milk	1.19 (0.96, 1.45)	0.97 (0.79, 1.20)	0.96 (0.77, 1.19)	1.01 (0.81, 1.25)
Cheese	0.82 (0.68, 0.99)^0.034^	0.95 (0.79, 1.15)	0.85 (0.70, 1.04)	0.66 (0.52, 0.83)^0.001^
Butter	1.07 (0.86, 1.34)	1.07 (0.86, 1.33)	0.95 (0.76, 1.19)	0.98 (0.77, 1.23)
B-Glucose				
Dairy products	1.10 (0.93, 1.30)	0.85 (0.70, 1.02)	1.02 (0.84, 1.23)	1.11 (0.89, 1.37)
Non-fermented milk	0.90 (0.75, 1.06)	0.98 (0.82, 1.16)	0.98 (0.82, 1.16)	0.87 (0.72, 1.06)
Fermented milk	0.92 (0.77, 1.09)	0.90 (0.76, 1.08)	0.89 (0.75, 1.06)	0.87 (0.72, 1.05)
Cheese	1.00 (0.85, 1.17)	0.96 (0.81, 1.14)	0.87 (0.73, 1.05)	0.90 (0.74, 1.09)
Butter	1.26 (1.04, 1.52)	1.11 (0.92, 1.34)	1.24 (1.03, 1.50)^0.027^	1.28 (1.04, 1.56)^0.018^
Blood pressure				
Dairy products	1.03 (0.92, 1.15)	1.03 (0.92, 1.15)	0.98 (0.87, 1.11)	0.99 (0.86, 1.14)
Non-fermented milk	1.04 (0.93, 1.15)	1.03 (0.93, 1.15)	0.93 (0.83, 1.05)	0.93 (0.82, 1.05)
Fermented milk	1.06 (0.95, 1.19)	1.07 (0.96, 1.19)	0.99 (0.88, 1.11)	0.93 (0.82, 1.04)
Cheese	1.00 (0.90, 1.11)	1.06 (0.95, 1.18)	1.08 (0.96, 1.21)	1.05 (0.93, 1.19)
Butter	0.91 (0.81, 1.02)	1.01 (0.90, 1.13)	0.99 (0.88, 1.11)	1.00 (0.88, 1.13)

Hazard ratio (95% CI) for developing an undesirable level of BMI, cholesterol, triglycerides, blood glucose, or blood pressure over the 8–12 year follow-up period and increasing quintile groups (Q1 to Q5) for intake of dairy products. Q1, lowest intake, is the reference category. Statistically significant *p*-values are in superscript

^a^Crude models with sex, age and dairy quintiles were tested and HRs were not statistically significant. The adjusted models included sex, age at follow-up, education, year at follow-up, BMI at follow up, physical activity at baseline and follow-up, mean intake of fruits and vegetables and energy over the follow-up period and dairy group. The models with overweight did not include BMI, and models including S-cholesterol or S-triglyceride were also adjusted for if the analyses were performed before or after the change of laboratory analyses methods

^b^Q1, Q2, Q3, Q4, Q5 median values (servings/day) are for all dairy products 1.6, 2.8, 3.9, 5.1, 7.1; for non-fermented milk 0.08, 0.58, 1.0, 1.4, 2.5; for fermented milk 0.006, 0.16, 0.36, 0.78, 1.1; for cheese 0.14, 0.36, 0.78, 1.0, 2.5; and for butter 0.003, 0.15, 1.0, 2.5, 3.2

### Prospective associations between dairy intake and cardiovascular risk factor deterioration

Individuals with desirable BMI, S-cholesterol, or S-triglycerides, fasting B-glucose or blood pressure at their first VIP visit and who maintained or deteriorated their status to an undesirable level over the follow-up period were identified from the subgroup of 27,682 individuals with a follow-up visit after 8–12 years (Fig. 1). In this prospective evaluation, increasing butter intake was associated with a deterioration of S-cholesterol from less than to above 5.2 mmol/l and of B-glucose from less than to above 6.1 mmol/l (Table 5). Compared to the reference group (Q1, i.e., lowest quintile for butter intake), the adjusted HR (95% CI) increased consecutively up to Q4 (1.16 (1.03, 1.30), *p* = 0.012) with stagnation for Q5 (1.12, (0.99, 1.26), *p* = 0.064). The HR for having an undesirable B-glucose at follow-up was 1.24 ((1.03, 1.50), *p* = 0.027) in Q4 and slightly higher in Q5 than in Q1 (Table 4). Further, increasing cheese intake was associated with a reduced risk of having undesirable S-triglyceride values at follow-up than at baseline. Thus, the HR (95% CI) for the Q5 group with the highest cheese intake was 0.66 ((0.52, 0.83), *p* = 0.001) compared to the Q1 group (Table 4). No associations were found for non-fermented or fermented milk or total dairy product intake.

### Characterization of subjects by reported dairy intake

**Fig. 2:**
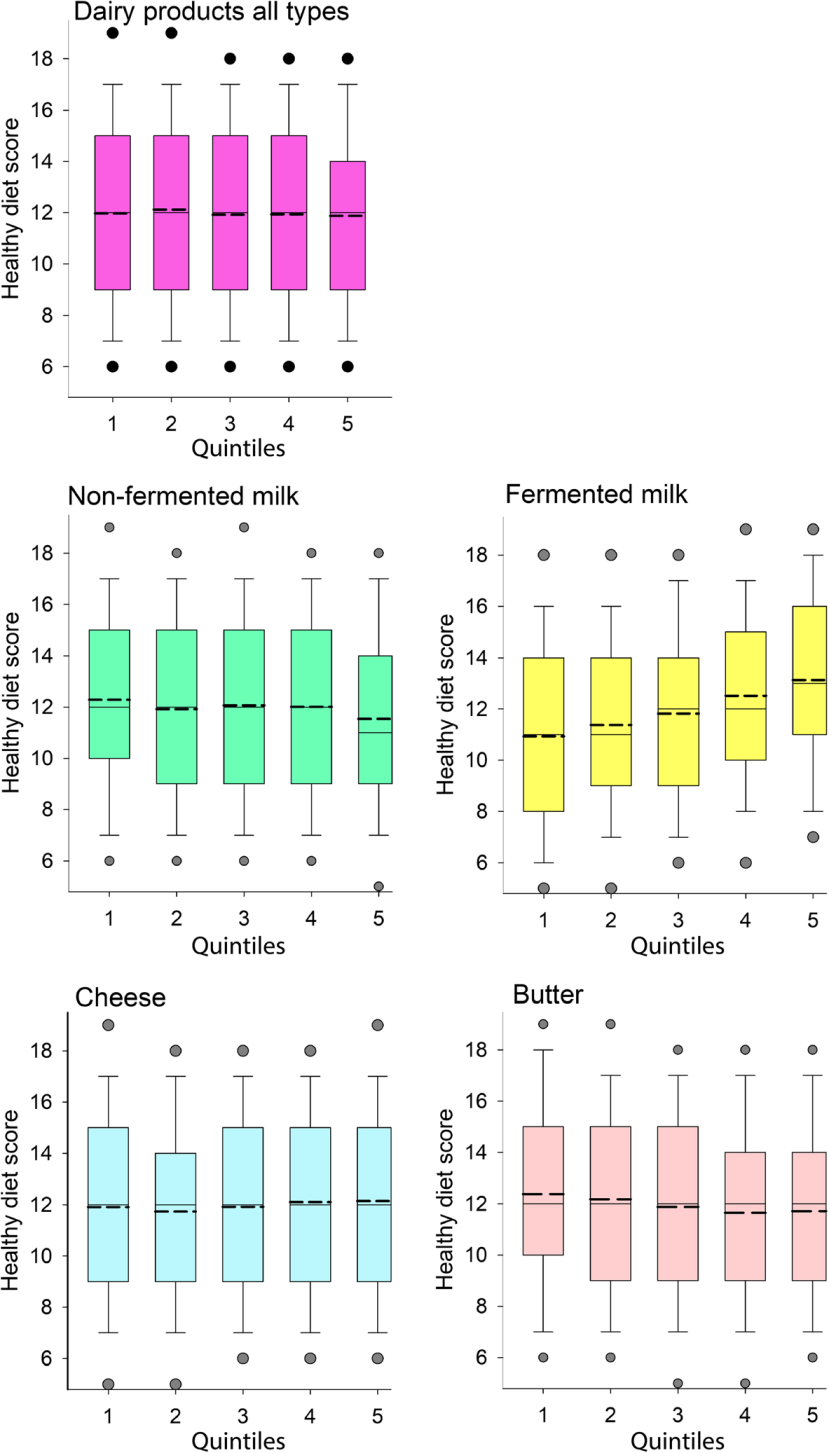
Healthy Diet Score by dairy intake. Box plots illustrating the median (straight line) and mean (dotted line) scores for quintile groups based on reported intake of total dairy products, non-fermented and fermented milk, cheese and butter. Whiskers represent the 25th and 75th percentile values, and dots represent the 5th and 95th percentile values

**Fig. 3:**
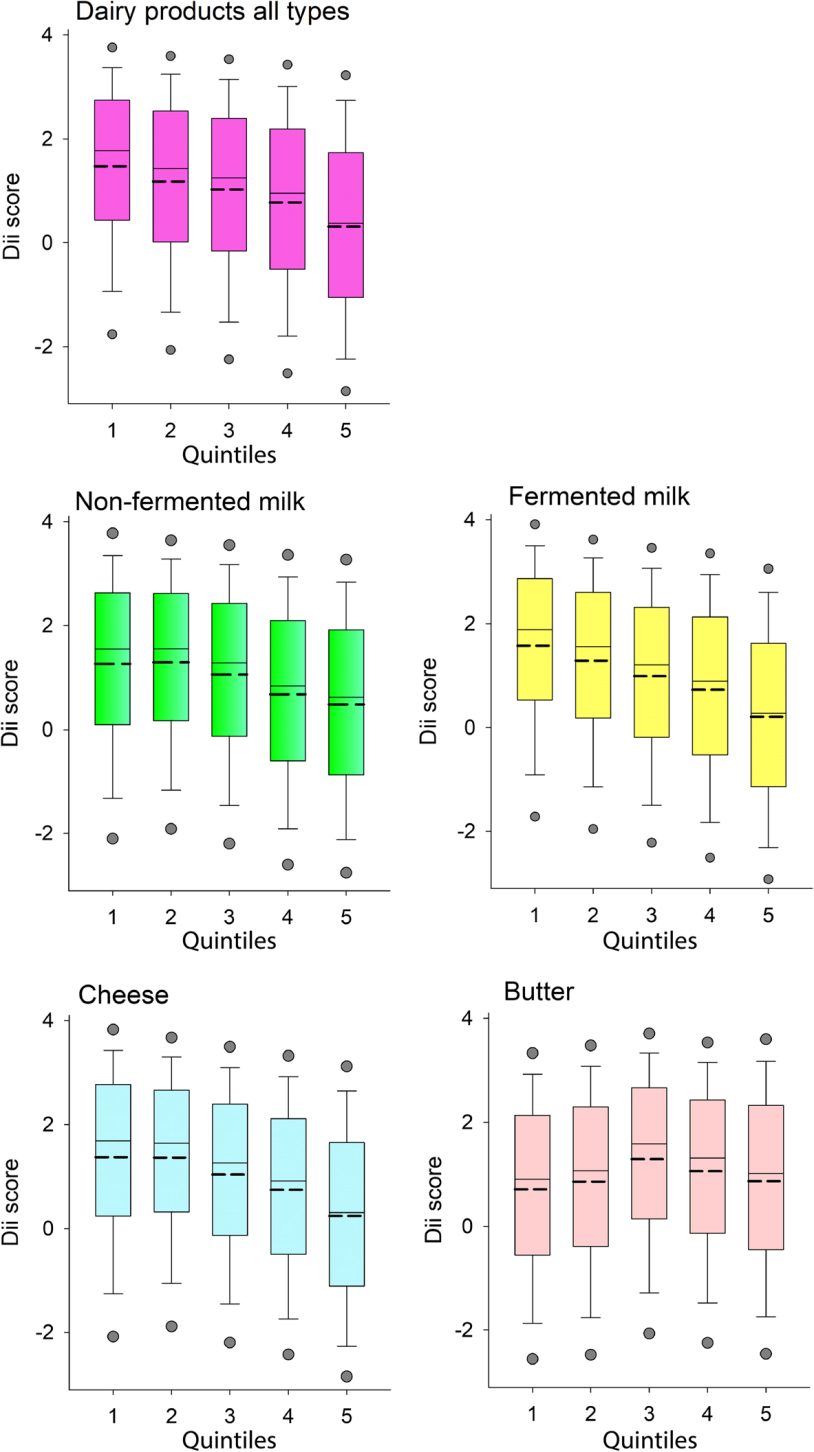
Diet Inflammation Index (DII) by dairy intake. Box plots illustrating the median (straight line) and mean (dotted line) scores for quintile groups based on reported intake of total dairy products, non-fermented and fermented milk, cheese and butter. Whiskers represent the 25th and 75th percentile values, and dots represent the 5th and 95th percentile values

To better understand the characteristics of the groups with different dairy intake, we evaluated their association with the scores from two diet indices that have been associated with health in other studies [29, 30]. These were the Healthy Diet Score with higher scores reflecting a healthier diet and an index reflecting the inflammatory potential of the diet (DII) with low scores reflecting a healthier diet. Overall, general linear modelling of sex, age, BMI and screening year adjusted means of the Healthy Diet Score increased with increasing intake of fermented milk and decreased for butter intake groups, whereas the DII score decreased consecutively with increasing dairy intake for all products except butter as shown in box plots for the quintile groups of dairy products (Figs. 2 and 3).

**Fig. 4:**
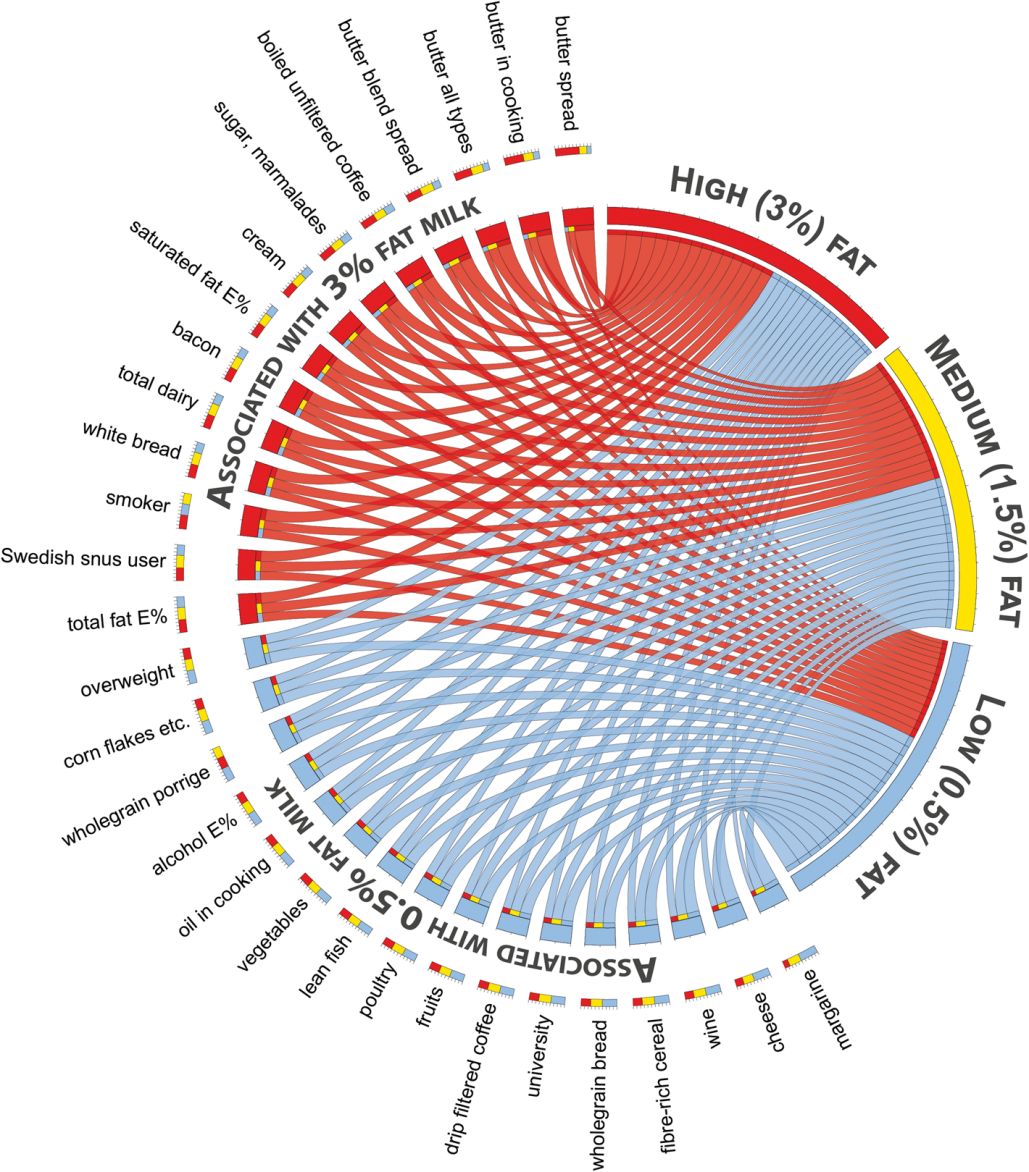
Circos plot for foods associated with non-fermented milk intake. The plot illustrates associations between cardiometabolic risk factors and sex, age, BMI, and reported energy adjusted intake of food items identified with a significant correlation with intake of non-fermented milk of different fat content. The lines illustrate the relationship between intake of high (3%) fat (red), medium (1.5%) fat (yellow), and low (0.5%) fat (blue) milk, respectively, and the outer circle reflects the proportion of the influential foods and cardiometabolic risk factors association with each of the three types of milk. Each food and cardiometabolic risk factor is color labeled as indicated above and sorted by strength of association to type of non-fermented milk consumed

**Fig. 5:**
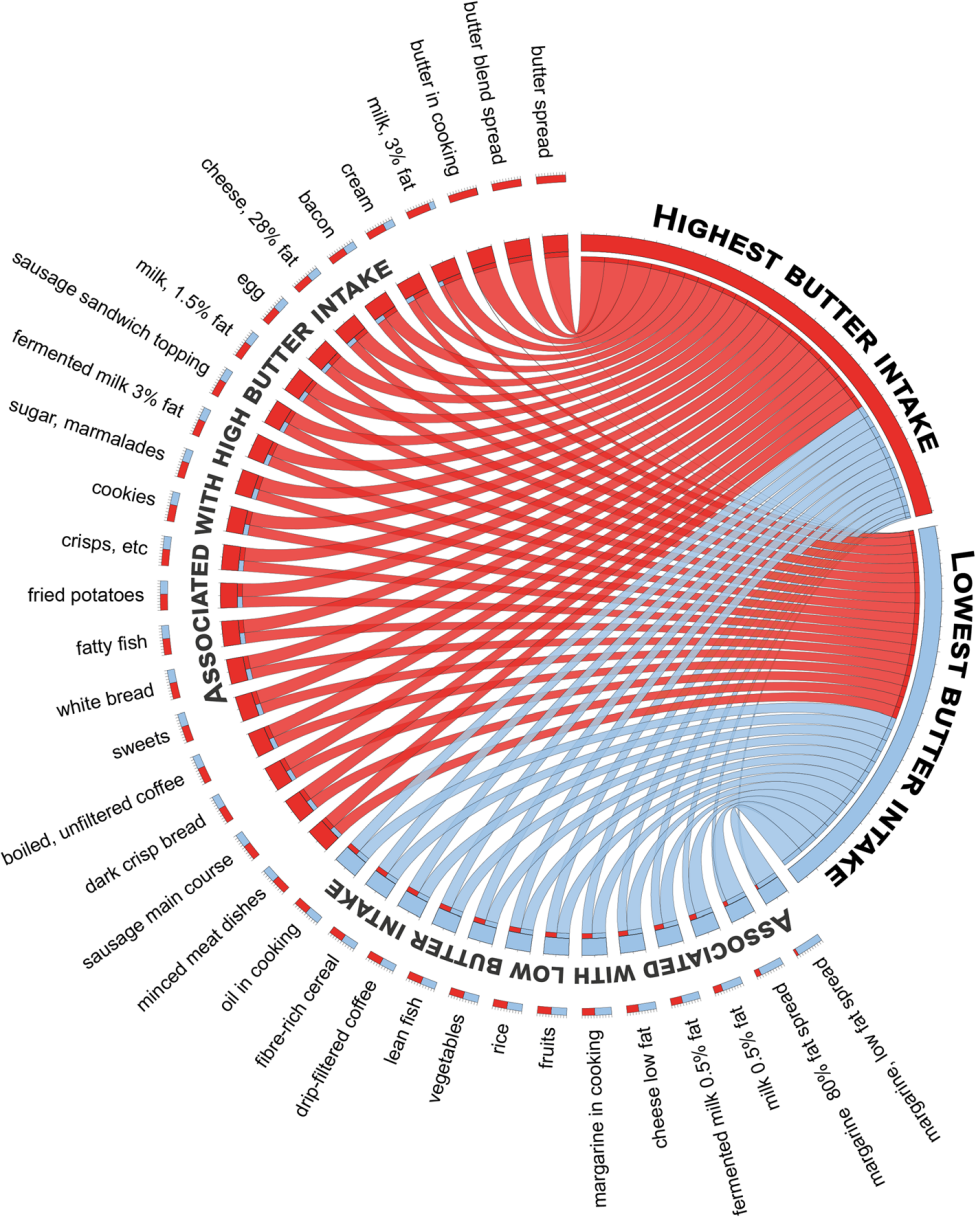
Circos plot for foods associated with highest or lowest butter intake. The plot illustrates associations between cardiometabolic risk factors and sex, age, BMI, and reported energy adjusted intake of food items identified with a significant correlation with intake of butter intake. The lines illustrate the relationship for those with the highest (red) versus lowest (blue) butter intake, respectively, and the outer circle reflects the proportion of the influential foods and cardiometabolic risk factors association with each of the butter intake levels. Each food and cardiometabolic risk factor is color labeled as indicated above and sorted by strength of association to butter consumption

We also compared the food selection patterns by PLS modelling as illustrated in Circos plots for subjects who reported exclusive intake, illustrating that high fat (3% fat) non-fermented milk consumers choose saturated fat or sugar products, whereas low (0.5% fat) fat non-fermented milk consumers choose foods that are considered health associated (Fig. 4). A similar pattern was seen when comparing those with the highest versus lowest butter intake (Fig. 5).

## Discussion

The present study follows three independent cohort studies from Sweden in which a positive association between non-fermented milk intake and all-cause mortality was reported [12, 14, 15] with an unclear nature of the association, i.e., causal or due to confounding bias. Here, a cross-sectional and longitudinal evaluation of associations between non-fermented milk and other dairy products and biological and lifestyle risk markers for cardiometabolic diseases [34] was performed in one of the three cohorts [14]. The study revealed an unfavourable profile of lifestyle factors in the present population, such as preference for butter, cream, sweet products, and use of tobacco products in high fat (3% fat) non-fermented milk and butter consumers. Further, some dairy products were associated with higher risk to have elevated cardio-metabolic risk factors, like BMI in high non-fermented milk consumers, and serum cholesterol in high consumers of total dairy products and butter, which, for butter, was confirmed in a prospective evaluation. These findings possibly support a contribution of confounding factors to the described association between total non-fermented milk intake and mortality in the same population [12, 14, 15].

In the present cohort, increasing reported intake of total dairy products and butter in both men and women and cheese in women was associated with decreased odds of having a BMI ≥ 25. Mean BMI was also lower in individuals who reported exclusive intake of high or middle fat non-fermented milk than in those with an intake of low fat non-fermented milk. These inverse associations have previously been reported in several cross-sectional studies [7, 36, 37]. However, Nordic and international dietary guidelines recommend low fat dairy intake for weight control [16, 17] and encourage overweight subjects to favour low fat dairy foods. Thus, the identified association may reflect reversal causality, a hypothesis supported by the fact that consumption of all types of dairy food variants exhibited no association with weight increase when analysed prospectively over 8–12 years. Further, three large-scale studies using genetically determined dairy intake as an instrumental variable in Mendelian randomization found higher consumption of total dairy [38] or non-fermented milk [39, 40] to be associated with significantly increased odds for overweight/obesity, supporting a causal association between consumption of these products and higher body weight.

The trend analysis for intake of total dairy and specific dairy groups showed that the intake of butter products increased continuously from 2007 onward. This finding was paralleled by an increased intake of high fat non-fermented milk (3% fat), whereas the intake of lower fat alternatives decreased. This trend is likely a result from the fatty-diet supporting trend in Swedish media for a period [19] combined with the inherent palatability of fatty and creamy foods [41]. Notably, choosing butter and high fat non-fermented milk was also associated with a panel of other less healthy food choices and worse scores on both healthy diet and inflammatory diet indices. Based on the present results with increased odds of having undesirable cholesterol levels (total and LDL) and hazard of deterioration of S-cholesterol levels over a decade, this trend seems unfortunate, and the impact on disease development must be monitored.

The strengths of the present study relate to the large study cohort with cross-sectional and longitudinal data in a population that is genetically and culturally homogenous for most of the participants. This strength is manifested in that 75% of participants reported an intake of non-fermented milk at least every third day and that 99% reported an intake of any dairy product every day. However, we cannot exclude some selection bias since the respondents had to be able to visit their local health clinic during working hours. However, validation efforts have not found any evidence for systematic bias [23, 24], and the large cohort likely compensates for random errors to some degree. Further, the well-known risk for measurement errors in diet intake data also exist here. Finally, there is an evident risk of reversed causality for BMI and dairy intake in cross-sectional analysis as discussed above, and residual confounding cannot be excluded.

Though several recent studies using genes as proxies for milk or dairy intake do not support a causal association with cardiometabolic risk factors or medical emergencies [2, 3, 38–40, 42], causal contributions cannot be fully excluded [43] and should be a target for further studies. However, the present results and those from other publications, support that confounding factors likely contribute to, and possibly explain, the demonstrated association between high dairy intake and unfavourable medical outcomes in populations that are similar to the one studied, leaving counselling on a healthy lifestyle overall as valid as ever. Importantly, type of diary product should be evaluated separately in future studies as these likely have different biological impacts and are proxies for different lifestyle patterns.

## Conclusions

Confounding factors likely contribute to the demonstrated association between dairy intake and mortality, and other medical conditions reported in populations like the one studied and the results support that future analyses should be stratified by dairy type.

## Additional files

Additional file 1: Reported dairy product intake presented as servings/day among all 29- to 65-year-old women and men at their first visit to the health screening. The study population lived in northern Sweden and data were collected from 1991 through 2016. Data are presented as means (95% CI limits) adjusted for BMI, estimated non-alcohol energy intake and screening year. Differences between age groups were tested with ANOVA in the general linear modeling procedure, and means differed significantly for all variables, i.e. all *p*-values < 0.001. (DOCX 34 kb)

Additional file 2: Odds ratio (95% CI limits) from multivariable logistic regression models for the association of being identified with an undesirable level of blood sugar (≥6.1 mmol/l) and increasing quintile groups (Q1 to Q5) for intake of dairy products. Q1, which represents the lowest intake, is the reference category. Statistically significant *p*-values are given in superscript. (DOCX 34 kb)

Additional file 3: Odds ratio (95% CI limits) from multivariable logistic regression models for the association of being classified with an undesirable level of blood pressure (defined as diastolic blood pressure ≥ 130 or systolic blood pressure ≥ 80) and increasing quintile groups (Q1 to Q5) for intake of dairy products. Q1, which represents the lowest intake, was the reference category. Statistically significant *p*-values are given in superscript. (DOCX 34 kb)

Additional file 4: Odds ratio (95% CI limits) from multivariable logistic regression models for the association of being classified with an undesirable level of HDL (defined as < 1 mmol/l) and increasing quintile groups (Q1 to Q5) for intake of dairy products. Data were collected from 2010 through 2016. Q1, which in the basic models represents the lowest intake and is the reference category. Statistically significant *p*-values are given in superscript. (DOCX 33 kb)

Additional file 5: Odds ratio (95% CI limits) from multivariable logistic regression models for the association of being classified with an undesirable level of LDL (defined as > 3 mmol/l) and increasing quintile groups (Q1 to Q5) for intake of dairy products. Data were collected from 2010 through 2016. Q1, which represents the lowest intake, was the reference category. Statistically significant *p*-values are given in superscript. (DOCX 32 kb)

## Abbreviations

BMI: Body Mass Index
DII: Dietary Inflammatory Index
FIL: Food Intake Level
HR: Hazard ratio
kCal: kilo calories
NSDD: the Northern Sweden Diet Database
NSHDS: The Northern Sweden Health and Disease Study
OR: Odds ratio
S-Cholesterol: Serum cholesterol
S-HDL: Serum high density lipoprotein
S-LDL: Serum low density lipoprotein
S-Triglycerides: Serum triglycerides
VIP: Västerbotten Intervention Program
