# Supplementary material for: Dairy intake revisited – associations between dairy intake and lifestyle related cardio-metabolic risk factors in a high milk consuming population
Source: Nutr J. 2018 Nov 22;17:110. doi: 10.1186/s12937-018-0418-y (PMC6251194; doi:10.1186/s12937-018-0418-y)
Supplement: Supplementary file 1 — Reported dairy product intake presented as servings/day among all 29- to 65-year-old women and men at their first visit to the health screening. The study population lived in northern Sweden and data were collected from 1991 through 2016. Data are presented as means (95% CI limits) adjusted for BMI, estimated non-alcohol energy intake and screening year. Differences between age groups were tested with ANOVA in the general linear modeling procedure, and means differed significantly for all variables, i.e. all p-values < 0.001. (DOCX 34 kb) [file 12937_2018_418_MOESM1_ESM.docx]

**Additional file 1.** Reported dairy product intake presented as servings/day among all 29- to 65-year-old women and men at their first visit to the health screening. The study population lived in northern Sweden and data were collected from 1991 through 2016. Data are presented as means (95% CI limits) adjusted for BMI, estimated non-alcohol energy intake and screening year. Differences between age groups were tested with ANOVA in the general linear modeling procedure, and means differed significantly for all variables, i.e. all p-values <0.001.

|  | **Servings/day** | | | |
| --- | --- | --- | --- | --- |
|  | **29-34 years**  3,823 women  3,597 men | **35-44 years**  21,176 women  19,943 men | **45-54 years**  12.936 women  12,723 men | **55-65 years**  8,527 women  7,787 men |
| **Total dairy products** |  |  |  |  |
| women | 3.42 (3.36, 3.48) | 3.90 (3.87, 3.92) | 3.99 (3.96, 4.02) | 4.56 (4.52, 4.59) |
| men | 3.67 (3.61, 3.73) | 4.35 (4.32, 4.38) | 4.34 (4.31, 4.37) | 4.48 (4.44, 4.52) |
| **Non-fermented milk** |  |  |  |  |
| women | 1.07 (1.04, 1.11) | 1.17 (1.16, 1.18) | 1.13 (1.11, 1.15) | 1.28 (1.26, 1.30) |
| men | 1.25 (1.22, 1.29) | 1.36 (1.35, 1.38) | 1.25 (1.24, 1.27) | 1.27 (1.25, 1.29) |
| **3% non-fermented milk** |  |  |  |  |
| women | 0.14 (0.13, 0.16) | 0.13 (0.12, 0.14) | 0.11 (0.11, 0.12) | 0.18 (0.17, 0.19) |
| men | 0.18 (0.16, 0.20) | 0.19 (0.18, 0.20) | 0.19 (0.18, 0.20) | 0.24 (0.23, 0.26) |
| **1.5% non-fermented milk** |  |  |  |  |
| women | 0.71 (0.68, 0.74) | 0.77 (0.76, 0.78) | 0.68 (0.66, 0.69) | 0.75 (0.73, 0.77) |
| men | 0.87 (0.84, 0.91) | 0.91 (0.90, 0.92) | 0.76 (0.74, 0.77) | 0.71 (0.69, 0.74) |
| **0.5% non-fermented milk** |  |  |  |  |
| women | 0.22 (0.20, 0.24) | 0.27 (0.26, 0.28) | 0.34 (0.33, 0.35) | 0.35 (0.33, 0.36) |
| men | 0.20 (0.18, 0.23) | 0.26 (0.25, 0.27) | 0.31 (0.30, 0.32) | 0.31 (0.29, 0.33) |
| **Fermented milk** |  |  |  |  |
| women | 0.46 (0.44, 0.48) | 0.58 (0.57, 0.58) | 0.66 (0.65, 0.67) | 0.77 (0.76, 0.78) |
| men | 0.37 (0.35, 0.39) | 0.51 (0.50, 0.51) | 0.54 (0.53, 0.55) | 0.58 (0.57, 0.59) |
| **3% fat fermented milk** |  |  |  |  |
| women | 0.30 (0.28, 0.31) | 0.37 (0.36, 0.37) | 0.39 (0.38, 0.40) | 0.46 (0.45, 0.47) |
| men | 0.27 (0.26, 0.28) | 0.36 (0.35, 0.36) | 0.37 (0.36, 0.37) | 0.37 (0.36, 0.38) |
| **0.5% fat fermented milk** |  |  |  |  |
| women | 0.16 (0.15, 0.18) | 0.21 (0.21, 0.22) | 0.27 (0.26, 0.27) | 0.32 (0.30, 0.32) |
| men | 0.10 (0.09, 0.11) | 0.15 (0.15, 0.15) | 0.17 (0.17, 0.18) | 0.21 (0.20, 0.22) |
| **Cheese** |  |  |  |  |
| women | 0.79 (0.77, 0.82) | 0.97 (0.96, 0.98) | 1.03 (1.02, 1.05) | 1.09 (1.08, 1.11) |
| men | 0.75 (0.72, 0.78) | 0.92 (0.91, 0.93) | 0.96 (0.95, 0.98) | 0.92 (0.90, 0.94) |
| **Cheese ≥28% fat** |  |  |  |  |
| Women | 0.57 (0.55, 0.60) | 0.69 (0.68, 0.70) | 0.72 (0.70, 0.73) | 0.72 (0.70, 0.73) |
| Men | 0.55 (0.52, 0.57) | 0.66 (0.65, 0.67) | 0.68 (0.67, 0.69) | 0.64 (0.62, 0.65) |
| **Cheese 10-17% fat** |  |  |  |  |
| women | 0.22 (0.20, 0.24) | 0.28 (0.27, 0.29) | 0.32 (0.31, 0.33) | 0.38 (0.37, 0.39) |
| men | 0.21 (0.19, 0.22) | 0.26 (0.25, 0.26) | 0.28 (0.27, 0.29) | 0.28 (0.27, 0.29) |
| **Butter** |  |  |  |  |
| women | 1.09 (1.05, 1.14) | 1.18 (1.17, 1.20) | 1.17 (1.15, 1.19) | 1.42 (1.39, 1.44) |
| men | 1.30 (1.25, 1.34) | 1.56 (1.54, 1.58) | 1.58 (1.56, 1.61) | 1.72 (1.68, 1.75) |
