# Supplementary material for: Dairy intake revisited – associations between dairy intake and lifestyle related cardio-metabolic risk factors in a high milk consuming population
Source: Nutr J. 2018 Nov 22;17:110. doi: 10.1186/s12937-018-0418-y (PMC6251194; doi:10.1186/s12937-018-0418-y)
Supplement: Supplementary file 2 — Odds ratio (95% CI limits) from multivariable logistic regression models for the association of being identified with an undesirable level of blood sugar (≥6.1 mmol/l) and increasing quintile groups (Q1 to Q5) for intake of dairy products. Q1, which represents the lowest intake, is the reference category. Statistically significant p-values are given in superscript. (DOCX 34 kb) [file 12937_2018_418_MOESM2_ESM.docx]

**Additional file 2.** Odds ratio (95% CI limits) from multivariable logistic regression models for the association of being identified with an undesirable level of **blood sugar** (≥6.1 mmol/l) and increasing quintile groups (Q1 to Q5) for intake of dairy products. Q1, which represents the lowest intake, is the reference category. Statistically significant p-values are given in superscript.

|  | Crude model (46,294 women and 43.899 men) | | | |  | Adjusted model (45,425 women and 43.094 men) | | | |
| --- | --- | --- | --- | --- | --- | --- | --- | --- | --- |
|  | Q2 | Q3 | Q4 | Q5 |  | Q2 | Q3 | Q4 | Q5 |
| Dairy products |  |  |  |  |  |  |  |  |  |
| women | 0.97 (0.88, 1.06) | 0.88 (0.80, 0.97)^0.008^ | 0.95 (0.87, 1.05) | 1.00 (0.91, 1.10) |  | 0.99 (0.89, 1.11) | 0.92 (0.81, 1.03) | 1.01 (0.90, 1.15) | 1.08 (0.95, 1.12) |
| men | 0.90 (0.82, 0.98)^0.016^ | 1.00 (0.91, 1.09) | 0.94 (0.86, 1.03) | 0.91 (0.83, 0.99)^0.034^ |  | 0.87(0.78, 0.96)^0.007^ | 0.99 (0.90, 1.11) | 0.91 (0.82, 1.02) | 0.91 (0.80, 1.04) |
| Non-fermented milk |  |  |  |  |  |  |  |  |  |
| women | 0.99 (0.89, 1.09) | 1.02 (0.92, 1.12) | 1.06 (0.96, 1.17) | 1.20 (1.09, 1.31)^<0.001^ |  | 0.96 (0.86, 1.08) | 1.02 (0.91, 1.15) | 1.06 (0.93, 1.20) | 1.16 (1.03, 1.31)^0.014^ |
| men | 1.04 (0.95, 1.14) | 0.99 (0.90, 1.08) | 1.05 (0.96, 1.15) | 1.14 (1.04, 1.24)^0.005^ |  | 0.99 (0.89, 1.10) | 0.94 (0.84, 1.04) | 1.00 (0.90, 1.11) | 1.06 (0.94, 1.19) |
| Fermented milk |  |  |  |  |  |  |  |  |  |
| women | 1.10 (1.01, 1.21)^0.037^ | 0.99 (0.90, 1.09) | 0.93 (0.84, 1.02) | 0.92 (0.83, 1.01) |  | 1.15 (1.03, 1.29)^0.014^ | 1.12 (0.99, 1.26) | 1.07 (0.95, 1.20) | 1.07 (0.94, 1.20) |
| men | 0.92 (0.84, 1.01) | 0.89 (0.82, 0.97)^0.011^ | 0.78(0.71, 0.86)^<0.001^ | 0.77 (0.71, 0.85)^<0.001^ |  | 0.92 (0.83, 1.03) | 0.95 (0.85, 1.06) | 0.89 (0.80, 1.00)^0.047^ | 0.92 (0.82, 1.03) |
| Cheese |  |  |  |  |  |  |  |  |  |
| women | 0.90 (0.82, 0.98)^0.018^ | 0.88 (0.81, 0.97)^0.007^ | 0.79(0.72, 0.87)^<0.001^ | 0.79 (0.72, 0.87)^<0.001^ |  | 0.89 (0.80, 0.99)^0.034^ | 0.94 (0.85, 1.05) | 0.86 (0.76, 0.96)^0.010^ | 0.86 (0.76, 0.98)^0.025^ |
| men | 1.06 (0.97, 1.16) | 0.95 (0.87, 1.04) | 0.99 (0.91, 1.08) | 0.95 (0.87, 1.04) |  | 1.08 (0.98, 1.20) | 0.96 (0.87, 1.07) | 1.03 (0.93, 1.15) | 0.99 (0.88, 1.11) |
| Butter |  |  |  |  |  |  |  |  |  |
| women | 0.89 (0.81, 0.98)^0.019^ | 0.99 (0.90, 1.09) | 0.94 (0.86, 1.04) | 0.95 (0.87, 1.05) |  | 0.98 (0.87, 1.10) | 1.04 (0.92, 1.16) | 1.00 (0.89, 1.12) | 0.98 (0.86, 1.10) |
| men | 0.95 (0.87, 1.04) | 0.93 (0.85, 1.02) | 0.92 (0.84, 1.00) | 0.93 (0.85, 1.01) |  | - 1. (0.83, 1.03) | 0.92 (0.83, 1.02) | 0.87 (0.78, 0.97)^0.011^ | 0.88 (0.78, 0.98)^0.022^ |

The crude models included age and dairy type. The adjusted models also included screening year, education, physical activity, BMI, smoking, and intakes of fruits and vegetables, alcohol and non-alcohol energy.
