# Supplementary material for: Dairy intake revisited – associations between dairy intake and lifestyle related cardio-metabolic risk factors in a high milk consuming population
Source: Nutr J. 2018 Nov 22;17:110. doi: 10.1186/s12937-018-0418-y (PMC6251194; doi:10.1186/s12937-018-0418-y)
Supplement: Supplementary file 3 — Odds ratio (95% CI limits) from multivariable logistic regression models for the association of being classified with an undesirable level of blood pressure (defined as diastolic blood pressure ≥ 130 or systolic blood pressure ≥ 80) and increasing quintile groups (Q1 to Q5) for intake of dairy products. Q1, which represents the lowest intake, was the reference category. Statistically significant p-values are given in superscript. (DOCX 34 kb) [file 12937_2018_418_MOESM3_ESM.docx]

**Additional file 3.** Odds ratio (95% CI limits) from multivariable logistic regression models for the association of being classified with an undesirable level of **blood pressure** (defined as diastolic blood pressure≥130 or systolic blood pressure ≥80) and increasing quintile groups (Q1 to Q5) for intake of dairy products. Q1, which represents the lowest intake, was the reference category. Statistically significant p-values are given in superscript.

|  | Crude model (46,105 women and 43,786 men) | | | |  | Adjusted model (45,237 women and 42,978 men) | | | |
| --- | --- | --- | --- | --- | --- | --- | --- | --- | --- |
|  | Q2 | Q3 | Q4 | Q5 |  | Q2 | Q3 | Q4 | Q5 |
| Dairy products |  |  |  |  |  |  |  |  |  |
| Women | 0.95 (0.89, 1.02) | 0.96 (0.90, 1.02) | 0.94 (0.88, 1.01) | 0.90 (0.84, 0.96)^0.001^ |  | 1.03 (0.95, 1.11) | 1.03 (0.94, 1.12) | 1.07 (0.98, 1.17) | 1.07 (0.96, 1.18) |
| Men | 0.91 (0.85, 0.96)^0.001^ | 0.91 (0.85, 0.96)^0.002^ | 0.88(0.83, 0.94)^<0.001^ | 0.88 (0.82, 0.93)^<0.001^ |  | 0.97 (0.90, 1.05) | 1.04 (0.96, 1.12) | 1.06 (0.97, 1.15) | 1.14 (1.04, 1.26)^0.005^ |
| Non-fermented milk |  |  |  |  |  |  |  |  |  |
| Women | 1.00 (0.94, 1.07) | 1.00 (0.94, 1.07) | 0.97 (0.90, 1.04) | 1.07 (1.00, 1.14)^0.047^ |  | 0.98 (0.90, 1.07) | 0.99 (0.91, 1.07) | 0.93 (0.85, 1.02) | 1.03 (0.94, 1.12) |
| Men | 0.96 (0.90, 1.02) | 0.95 (0.89, 1.01) | 0.90 (0.85, 0.96)^<0.001^ | 0.93 (0.87, 0.99)^0.016^ |  | 0.95 (0.88, 1.03) | 1.01 (0.94, 1.09) | 0.96 (0.89, 1.04) | 1.02 (0.94, 1.11) |
| Fermented milk |  |  |  |  |  |  |  |  |  |
| Women | 0.98 (0.92, 1.05) | 0.98 (0.91, 1.04) | 0.91 (0.85, 0.97)^0.003^ | 0.89 (0.83, 0.95)^<0.001^ |  | 0.93 (0.86, 1.01) | 0.96 (0.88, 1.04) | 0.91 (0.84, 0.99)^0.027^ | 0.92 (0.84, 1.00)^0.051^ |
| Men | 0.92 (0.87, 0.98)^0.008^ | 0.88(0.82, 0.93)^<0.001^ | 0.81(0.76, 0.86)^<0.001^ | 0.78 (0.73, 0.82)^<0.001^ |  | 0.91 (0.85, 0.99)^0.024^ | 0.87 (0.81, 0.95)^0.001^ | 0.83 (0.77, 0.90)^<0.001^ | 0.88 (0.81, 0.95)^0.002^ |
| Cheese |  |  |  |  |  |  |  |  |  |
| Women | 1.01 (0.95, 1.08) | 0.96 (0.90, 1.02) | 0.95 (0.88, 1.01) | 0.90 (0.84, 0.96)^0.001^ |  | 1.01 (0.94, 1.09) | 0.98 (0.90, 1.06) | 1.06 (0.97, 1.15) | 0.95 (0.86, 1.04) |
| Men | 1.01 (0.95, 1.07) | 0.92 (0.87, 0.98)^0.010^ | 0.86 (0.81, 0.91)^<0.001^ | 0.87 (0.82, 0.93)^<0.001^ |  | 1.05 (0.98, 1.13) | 0.99 (0.91, 1.07) | 1.00 (0.92, 1.08) | 1.04 (0.96, 1.13) |
| Butter |  |  |  |  |  |  |  |  |  |
| Women | 0.94 (0.88, 1.00)^0.045^ | 0.91 (0.85, 0.97)^0.004^ | 0.94 (0.89, 1.01) | 0.85 (0.80, 0.91)^<0.001^ |  | 0.94 (0.86, 1.02) | 0.94 (0.87, 1.03) | 1.03 (0.94, 1.12) | 1.00 (0.92, 1.09) |
| Men | 1.02 (0.96, 1.09) | 1.06 (1.00, 1.13) | 1.00 (0.94, 1.06) | 1.02 (0.96, 1.08) |  | 1.03 (0.96, 1.11) | 1.01 (0.94, 1.09) | 1.01 (0.93, 1.09) | 1.04 (0.96, 1.13) |

The crude models included age and dairy type. The adjusted models also included screening year, education, physical activity, BMI, smoking, and intakes of fruits and vegetables, alcohol and non-alcohol energy.
