# Supplementary material for: Dairy intake revisited – associations between dairy intake and lifestyle related cardio-metabolic risk factors in a high milk consuming population
Source: Nutr J. 2018 Nov 22;17:110. doi: 10.1186/s12937-018-0418-y (PMC6251194; doi:10.1186/s12937-018-0418-y)
Supplement: Supplementary file 4 — Odds ratio (95% CI limits) from multivariable logistic regression models for the association of being classified with an undesirable level of HDL (defined as < 1 mmol/l) and increasing quintile groups (Q1 to Q5) for intake of dairy products. Data were collected from 2010 through 2016. Q1, which in the basic models represents the lowest intake and is the reference category. Statistically significant p-values are given in superscript. (DOCX 33 kb) [file 12937_2018_418_MOESM4_ESM.docx]

**Additional file 4.** Odds ratio (95% CI limits) from multivariable logistic regression models for the association of being classified with an undesirable level of **HDL** (defined as <1 mmol/l) and increasing quintile groups (Q1 to Q5) for intake of dairy products. Data were collected from 2010 through 2016. Q1, which in the basic models represents the lowest intake and is the reference category. Statistically significant p-values are given in superscript.

|  | Crude model (8,398 women and 8,496 men) | | | |  | Adjusted model (8,165 women and 8,236 men) | | | |  |
| --- | --- | --- | --- | --- | --- | --- | --- | --- | --- | --- |
|  | Q2 | Q3 | Q4 | Q5 |  | Q2 | Q3 | Q4 | Q5 | |
| Dairy products |  |  |  |  |  |  |  |  |  | |
| women | 0.75 (0.55, 1.03) | 0.81 (0.59, 1.10) | 0.78 (0.57, 1.07) | 0.70 (0.51, 0.96)^0.029^ |  | 0.79 (0.56, 1.11) | 0.86 (0.61, 1.19) | 0.79 (0.56, 1.12) | 0.72 (0.48, 1.07) | |
| men | 0.84 (0.71 ,0.99)^0.034^ | 0.86 (0.73, 1.02) | 0.65(0.55, 0.77)^<0.001^ | 0.79 (0.67, 0.94)^0.007^ |  | 0.79 (0.66, 0.94)^0.007^ | 0.82 (0.68, 0.99)^0.035^ | 0.59 (0.48, 0.73)^<0.001^ | 0.69 (0.55, 0.86)^0.001^ | |
| Non-fermented milk |  |  |  |  |  |  |  |  |  | |
| women | 0.99 (0.73, 1.34) | 0.98 (0.72, 1.34) | 0.99 (0.69, 1.41) | 1.19 0.88, 1.61) |  | 0.93 (0.68, 1.27) | 0.94 (0.68, 1.31) | 0.97 (0.67, 1.41) | 1.04 (0.74, 1.45) | |
| men | 1.11 (0.95 ,1.30) | 0.99 (0.83, 1.17) | 0.96 (0.81, 1.13) | 1.22 (1.02, 1.44)^0.026^ |  | 1.12 (0.94, 1.33) | 1.04 (0.87, 1.25) | 0.95 (0.79, 1.14) | 1.15 (0.95, 1.40) | |
| Fermented milk |  |  |  |  |  |  |  |  |  | |
| women | 0.97 (0.71, 1.32) | 0.96 (0.70, 1.31) | 0.80 (0.58, 1.10) | 0.84 (0.61, 1.16) |  | 0.91 (0.66, 1.26) | 1.01 (0.73, 1.41) | 0.82 (0.58, 1.16) | 0.96 (0.68, 1.35) | |
| men | 0.88 (0.75 ,1.04) | 0.87 (0.73, 1.03) | 0.76 (0.64, 0.90)^0.002^ | 0.73 (0.62, 0.87)^0.001^ |  | 0.94 (0.78, 1.13) | 0.96 (0.80, 1.15) | 0.90 (0.75, 1.09) | 0.87 (0.72, 1.06) | |
| Cheese |  |  |  |  |  |  |  |  |  | |
| women | 1.05 (0.81, 1.37) | 0.87 (0.65, 1.17) | 0.88 (0.60, 1.29) | 0.73 (0.49, 1.08) |  | 1.09 (0.82, 1.45) | 0.98 (0.71, 1.34) | 1.01 (0.68, 1.52) | 0.86 (0.56, 1.32) | |
| men | 0.92 (0.79 ,1.07) | 0.86 (0.73, 1.00) | 0.73 (0.61, 0.86)^<0.001^ | 0.77 (0.64, 0.92)^0.005^ |  | 0.93 (0.79, 1.10) | 0.87 (0.73, 1.04) | 0.75 (0.62, 0.91)^0.003^ | 0.76 (0.62, 0.94)^0.010^ | |
| Butter |  |  |  |  |  |  |  |  |  | |
| women | 0.78(0.62, 1.19) | 0.77 (0.54, 1.11) | 0.68 (0.47, 0.97)^0.034^ | 0.53 (0.36, 0.77)^0.001^ |  | 0.88 (0.57, 1.38) | 0.86 (0.58, 1.27) | 0.74 (0.50, 1.09) | 0.61 (0.41, 0.92)^0.019^ | |
| men | 0.84 (0.68, 1.02) | 0.74 (0.61, 0.89)^0.002^ | 0.76(0.61, 0.93)^0.009^ | 0.66 (0.54, 0.80)^<0.001^ |  | 0.89 (0.72, 1.11) | 0.82 (0.67, 1.00) | 0.80 (0.64, 1.01) | 0.67(0.54, 0.84)^<0.001^ | |

The crude models included age and dairy type. The adjusted models also included education, physical activity, BMI, smoking, screening year and intakes of fruits and vegetables, alcohol and non-alcohol energy.
