# Supplementary material for: Dairy intake revisited – associations between dairy intake and lifestyle related cardio-metabolic risk factors in a high milk consuming population
Source: Nutr J. 2018 Nov 22;17:110. doi: 10.1186/s12937-018-0418-y (PMC6251194; doi:10.1186/s12937-018-0418-y)
Supplement: Supplementary file 5 — Odds ratio (95% CI limits) from multivariable logistic regression models for the association of being classified with an undesirable level of LDL (defined as > 3 mmol/l) and increasing quintile groups (Q1 to Q5) for intake of dairy products. Data were collected from 2010 through 2016. Q1, which represents the lowest intake, was the reference category. Statistically significant p-values are given in superscript. (DOCX 32 kb) [file 12937_2018_418_MOESM5_ESM.docx]

**Additional file 5.** Odds ratio (95% CI limits) from multivariable logistic regression models for the association of being classified with an undesirable level of **LDL** (defined as >3 mmol/l) and increasing quintile groups (Q1 to Q5) for intake of dairy products. Data were collected from 2010 through 2016. Q1, which represents the lowest intake, was the reference category. Statistically significant p-values are given in superscript.

|  | Crude model (8,369 women and 8,287 men) | | | |  | Adjusted model (8,137 women and 8,037 men) | | | |  |
| --- | --- | --- | --- | --- | --- | --- | --- | --- | --- | --- |
|  | Q2 | Q3 | Q4 | Q5 |  | Q2 | Q3 | Q4 | Q5 | |
| Dairy products |  |  |  |  |  |  |  |  |  | |
| women | 0.97 (0.84, 1.12) | 1.11 (0.96, 1.28) | 1.01 (0.87, 1.16) | 1.02 (0.89, 1.18) |  | 1.02 (0.87, 1.18) | 1.16 (0.99, 1.35) | 1.06 (0.90, 1.24) | 1.15 (0.96, 1.37) | |
| men | 1.02 (0.88, 1.18) | 1.10 (0.94, 1.27) | 1.06 (0.91, 1.23) | 1.14 (0.98, 1.32) |  | 1.05 (0.91, 1.23) | 1.14 (0.97, 1.34) | 1.17 (0.99, 1.38) | 1.31 (1.08, 1.58)^0.006^ | |
| Non-fermented milk |  |  |  |  |  |  |  |  |  | |
| women | 1.04 (0.91, 1.18) | 1.05 (0.92, 1.20) | 1.00 (0.86, 1.17) | 1.05 (0.92, 1.21) |  | 1.00 (0.87, 1.14) | 1.03 (0.90, 1.19) | 1.00 (0.85, 1.18) | 0.99 (0.85, 1.15) | |
| men | 1.12 (0.97, 1.28) | 1.09 (0.94, 1.26) | 1.05 (0.91, 1.21) | 1.17 (1.00, 1.36)^0.045^ |  | 1.12 (0.97, 1.30) | 1.12 (0.96, 1.30) | 1.07 (0.92, 1.25) | 1.15 (0.97, 1.36) | |
| Fermented milk |  |  |  |  |  |  |  |  |  | |
| women | 1.19 (1.03, 1.36)^0.016^ | 1.10 (0.96, 1.27) | 1.05 (0.92, 1.21) | 0.97 (0.84, 1.11) |  | 1.16(1.00, 1.34) | 1.16 (1.00, 1.35) | 1.13 (0.98, 1.32) | 1.06 (0.91, 1.24) | |
| men | 0.98 (0.84, 1.14) | 1.03 (0.89, 1.20) | 0.92 (0.79, 1.07) | 0.86 (0.74, 1.00) |  | 1.01 (0.86, 1.18) | 1.08 (0.92, 1.26) | 1.03 (0.88, 1.21) | 0.97 (0.83, 1.15) | |
| Cheese |  |  |  |  |  |  |  |  |  | |
| women | 1.11 (0.98, 1.25) | 1.14 (1.01, 1.30)^0.041^ | 1.09 (0.93, 1.29) | 0.88 (0.75, 1.03) |  | 1.11 (0.97, 1.26) | 1.16 (1.01, 1,33)^0.036^ | 1.06 (0.89, 1.26) | 0.94 (0.79, 1.12) | |
| men | 1.08 (0.94, 1.24) | 1.00 (0.87, 1.15) | 0.91 (0.79, 1.06) | 0.90 (0.77, 1.06) |  | 1.10 (0.95, 1.27) | 1.00 (0.87, 1.16) | 0.94 (0.80, 1.09) | 0.93 (0.78, 1.10) | |
| Butter |  |  |  |  |  |  |  |  |  | |
| women | 1.02 (0.83, 1.25) | 1.19 (0.99, 1.42) | 1.20 (1.00, 1.43) | 1.16 (0.97, 1.38) |  | 1.07 (0.86, 1.33) | 1.21 (1.00, 1.47)^0.049^ | 1.28 (1.06, 1.55)^0.011^ | 1.28 (1.05,1.56)^0.013^ | |
| men | 1.22 (1.02, 1.46)^0.029^ | 1.47 (1.24, 1.74)^<0.001^ | 1.43(1.19, 1.73)^<0.001^ | 1.48 (1.25, 1.76)^<0.001^ |  | 1.23(1.03, 1.49)^0.026^ | 1.48(1.24, 1.77)^<0.001^ | 1.49(1.22, 1.81)^<0.001^ | 1.62(1.35, 1.96)^<0.001^ | |

The crude models included age and dairy type. The adjusted models also included education, physical activity, BMI, smoking, screening year and intakes of fruits and vegetables, alcohol and non-alcohol energy.
